# Supplementary material for: The added benefit of including cognitive coping in brief psychosocial interventions: A randomized controlled trial among veterans and family members in Ukraine
Source: Glob Ment Health (Camb). 2025 Oct 7;12:e111. doi: 10.1017/gmh.2025.10065 (PMC12538515; doi:10.1017/gmh.2025.10065)

**Supplemental Table 1. Sample description throughout study progression**				
	**All Registration Data** ***(n=2548)***	**Consented Study Sample** ***(n=1177)***	**Follow-Up/ Analytic Sample** ***(n=788)***	**Aim 3 Referral Sample** ***(n=383)***
**Vet Status**				
Veteran/Volunteer	54.2%	48.2%	45.4%	52.5%
Family member of vet	45.8%	51.8%	54.6%	47.5%
**Gender**				
Male	44.8%	41.0%	40.5%	44.9%
Female	54.9%	58.9%	59.4%	55.1%
Missing	0.27%	0.1%	.1%	--
**Age** (Mean, SD)	40.2 (10.5)	40.7 (10.6)	40.7 (10.5)	41.2 (10.1)
**Referral Source**				
Facebook	23.6%	15.4%	17.3%	23.8%
Family	3.2%	3.5%	3.1%	3.4%
Friend	19.7%	18.0%	15.5%	16.5%
Provider	26.7%	35.5%	38.2%	25.6%
Other	21.1%	22.9%	21.6%	24.8%
Missing	5.7%	4.7%	4.4%	6.0%
**Motivation***	**(n=2126)**	**(n=1030)**	**(n=672)**	**(n=306)**
Learn about stress	40.4%	46.7%	48.7%	40.9%
Deal with stress	61.2%	58.8%	56.3%	65.7%
Deal with emotions	51.8%	51.2%	48.4%	57.5%
Deal with aggression	34.0%	32.1%	30.8%	37.9%
Learn where to get help	19.6%	24.0%	26.6%	24.5%
Help others cope	29.0%	26.6%	25.6%	30.1%
Help others get help	15.6%	14.7%	15.5%	17.0%
Someone wanted me to come	3.9%	3.2%	3.0%	3.9%
Other	2.8%	2.3%	2.1%	2.6%
*Motivation was initially collected as free response (so smaller *n* for tabulated responses)				

Supplemental Figure 1. Effect Size for Distress Calculated With Iterative Removal and Replacement of Each Provider Pair

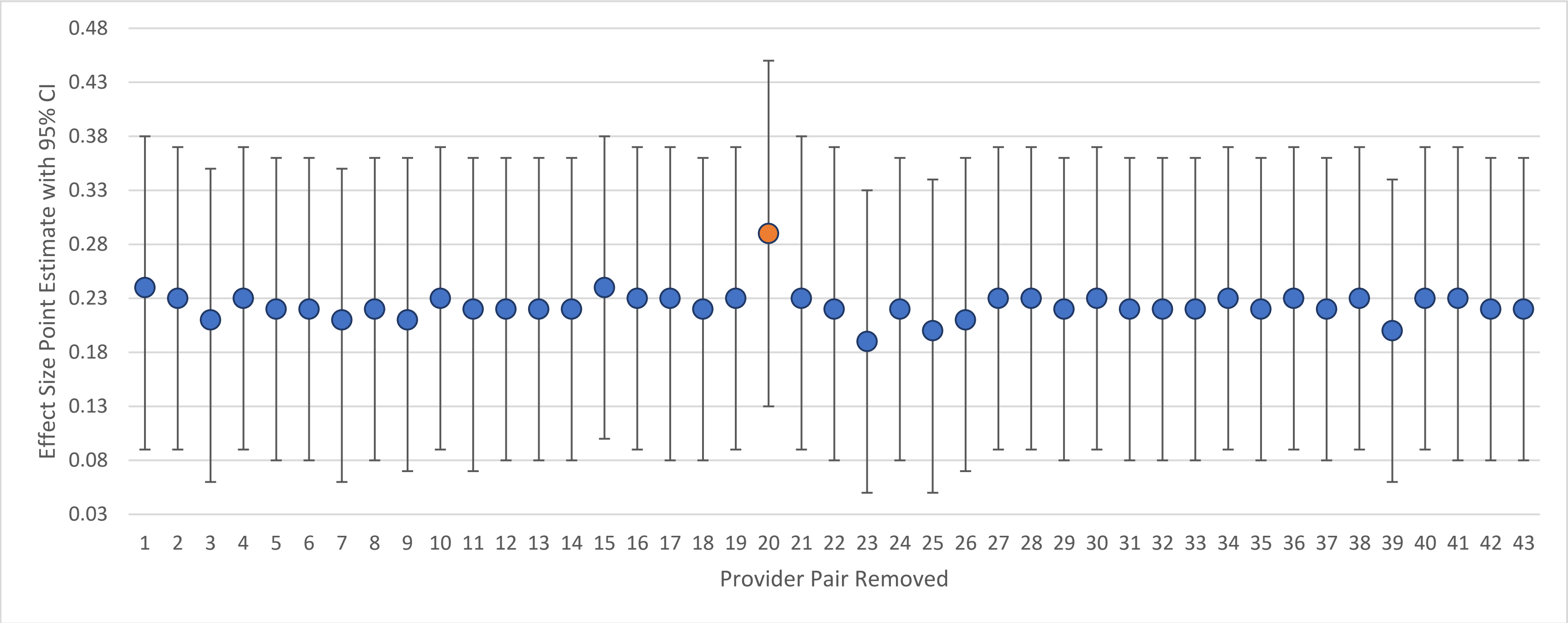

Supplemental Figure 2. Consort Diagram for Sensitivity Analysis

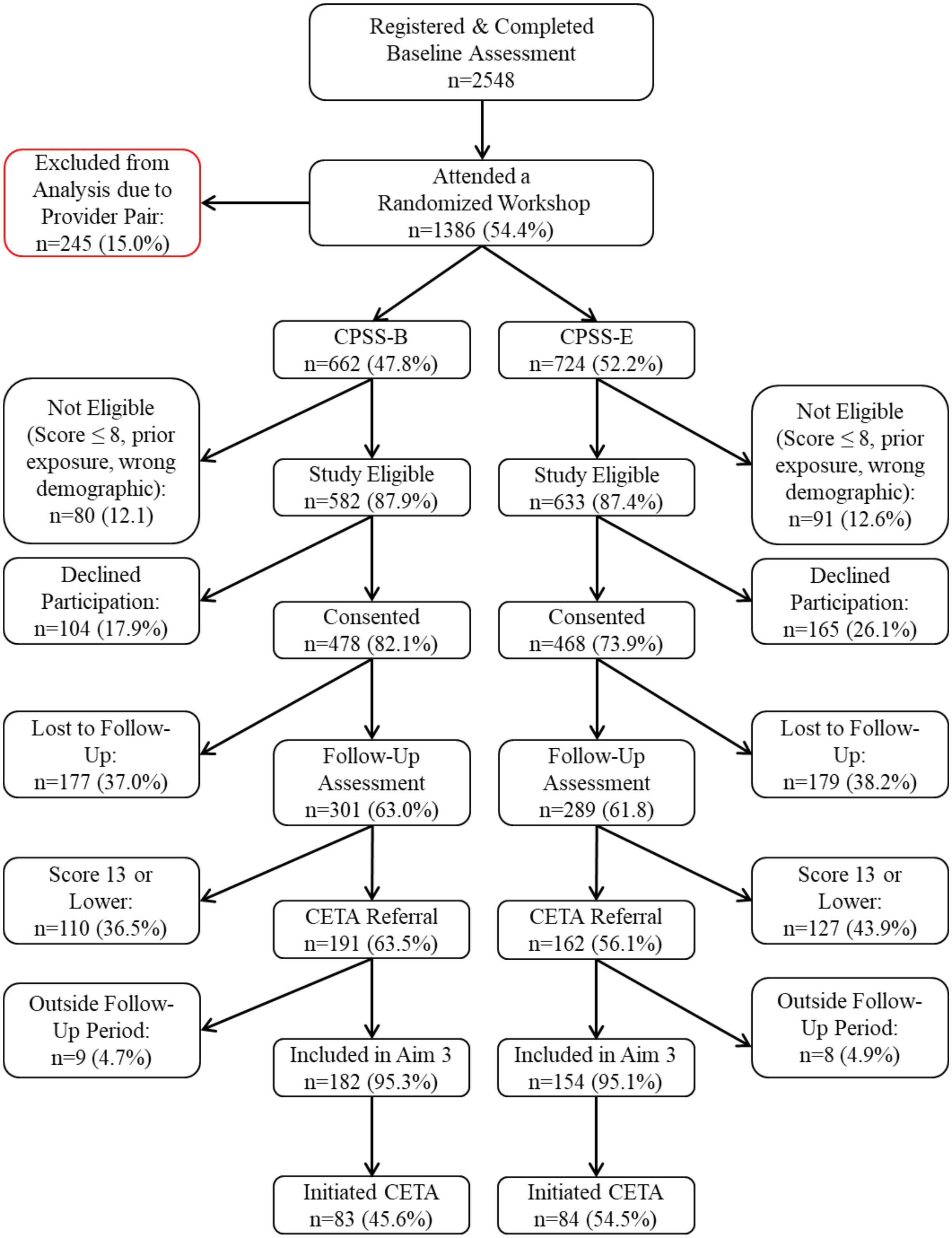

Supplement: Nguyen et al. supplementary material [file S2054425125100654sup001.pdf]
